# *In crystallo* screening for proline analog inhibitors of the proline cycle enzyme PYCR1

**DOI:** 10.1074/jbc.RA120.016106

**Published:** 2021-01-13

**Authors:** Emily M. Christensen, Alexandra N. Bogner, Anke Vandekeere, Gabriela S. Tam, Sagar M. Patel, Donald F. Becker, Sarah-Maria Fendt, John J. Tanner

**Affiliations:** 1 Department of Chemistry, University of Missouri, Columbia, Missouri, USA; 2 Department of Biochemistry, University of Missouri, Columbia, Missouri, USA; 3 Laboratory of Cellular Metabolism and Metabolic Regulation, VIB-KU Leuven Center for Cancer Biology, VIB, Leuven, Belgium; 4 Department of Oncology, Laboratory of Cellular Metabolism and Metabolic Regulation, KU Leuven and Leuven Cancer Institute (LKI), Leuven, Belgium; 5 Department of Biochemistry, Redox Biology Center, University of Nebraska, Lincoln, Nebraska, USA

**Keywords:** X-ray crystallography, enzyme inhibitor, enzyme kinetics, breast cancer, tumor metabolism

## Abstract

Pyrroline-5-carboxylate reductase 1 (PYCR1) catalyzes the biosynthetic half-reaction of the proline cycle by reducing Δ^1^-pyrroline-5-carboxylate (P5C) to proline through the oxidation of NAD(P)H. Many cancers alter their proline metabolism by up-regulating the proline cycle and proline biosynthesis, and knockdowns of PYCR1 lead to decreased cell proliferation. Thus, evidence is growing for PYCR1 as a potential cancer therapy target. Inhibitors of cancer targets are useful as chemical probes for studying cancer mechanisms and starting compounds for drug discovery; however, there is a notable lack of validated inhibitors for PYCR1. To fill this gap, we performed a small-scale focused screen of proline analogs using X-ray crystallography. Five inhibitors of human PYCR1 were discovered: l-tetrahydro-2-furoic acid, cyclopentanecarboxylate, l-thiazolidine-4-carboxylate, l-thiazolidine-2-carboxylate, and *N*-formyl l-proline (NFLP). The most potent inhibitor was NFLP, which had a competitive (with P5C) inhibition constant of 100 μm. The structure of PYCR1 complexed with NFLP shows that inhibitor binding is accompanied by conformational changes in the active site, including the translation of an α-helix by 1 Å. These changes are unique to NFLP and enable additional hydrogen bonds with the enzyme. NFLP was also shown to phenocopy the *PYCR1* knockdown in MCF10A H-RAS^V12^ breast cancer cells by inhibiting *de novo* proline biosynthesis and impairing spheroidal growth. In summary, we generated the first validated chemical probe of PYCR1 and demonstrated proof-of-concept for screening proline analogs to discover inhibitors of the proline cycle.

Proline metabolism has attracted interest recently because of its involvement in the metabolic rewiring that occurs in cancer cells (1, 2). A unique function of proline metabolism in cancer is the enzymatic cycling of proline and Δ^1^-pyrroline-5-carboxylate (P5C), known as the proline cycle (Fig. 1). The catabolic half-reaction of the proline cycle is the oxidation of proline to P5C catalyzed by the flavoenzyme proline dehydrogenase (PRODH) in mitochondria. The biosynthetic half-reaction is the reduction of P5C to proline catalyzed by NAD(P)H-dependent P5C reductase isoform 1 (PYCR1) (Fig. 1). These linked half-cycles provide a mechanism for maintaining pyridine nucleotide levels, generating reactive oxygen species, and producing ATP (3, 4). This is particularly relevant in metastases, where both PRODH and PYCR1 have been shown to be up-regulated in models of breast cancer metastasis (5). Mechanistically, the increased flux through the proline cycle allows for the amplified production of ATP by PRODH at the expense of the PYCR1 cofactor NAD(P)H, which supports colonization and formation of secondary tumors at distant organs (5). Thus, the proline cycle enzymes PRODH and PYCR1 are emerging cancer therapy targets (6).

**Figure 1:**
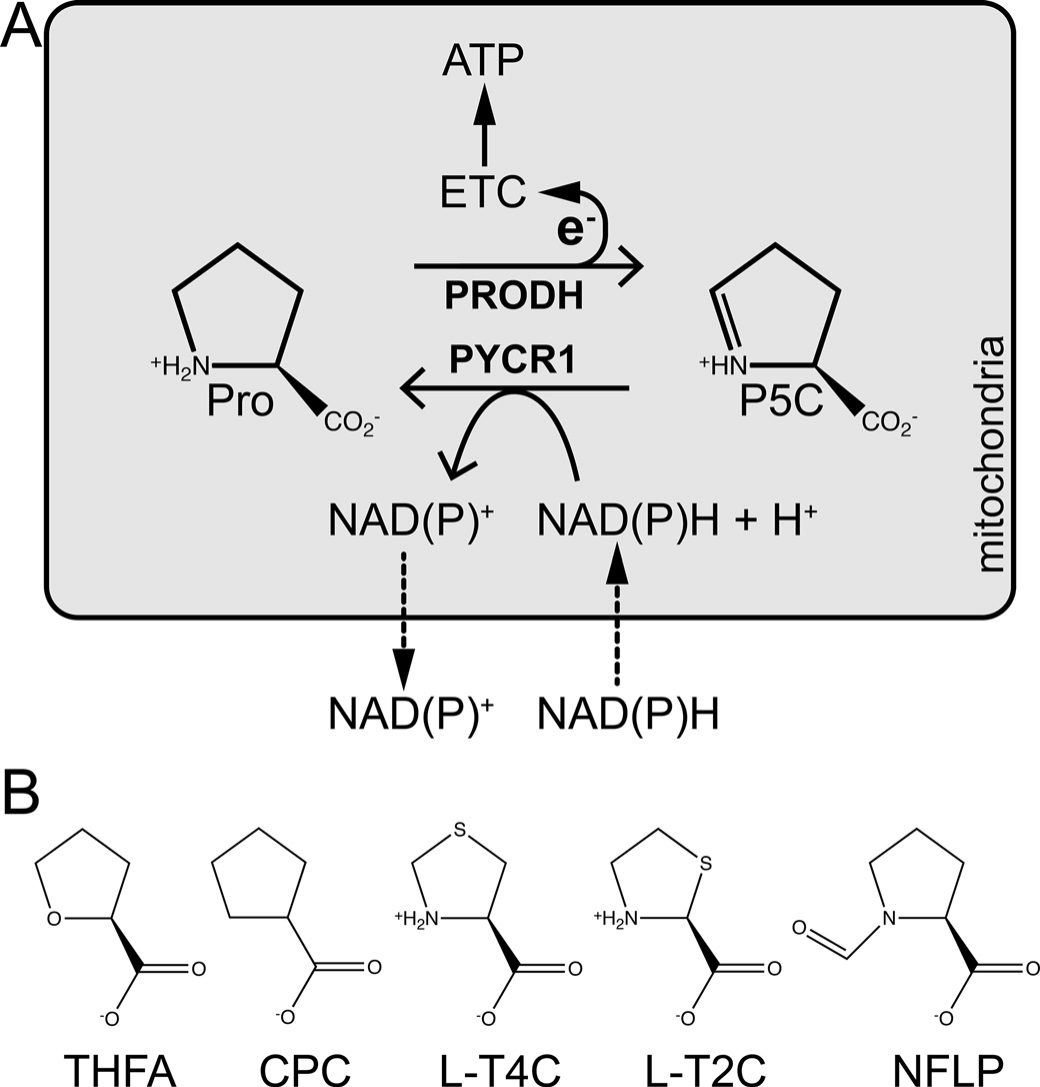
**The proline cycle and inhibitors of proline cycle enzymes.***A*, the enzymes and reactions of the proline cycle. *B*, chemical structures of THFA, CPC, l-T4C, l-T2C, and NFLP.

An important role for PYCR1 in cancer is also suggested by numerous studies showing that it is one of the most consistently overexpressed metabolic enzymes across cancer types (7). Accordingly, knockdowns of PYCR1 have resulted in decreased cell proliferation in kidney cancer (8), lung carcinoma (9), and liver cancer (10). The *PYCR1* gene has also been linked to multiple cellular capabilities arising from metabolic reprogramming in cancer, including clonogenicity (11), invasiveness (12), and metastatic seeding (5). Furthermore, because of the antioxidant capacity of proline, overexpression of PYCR1 and increased proline biosynthesis may contribute to enhanced cancer cell survival (13, 14, 15, 16). Likewise, a recent study of wound healing showed that the induction of proline biosynthesis protects fibroblasts from the damaging effects of transforming growth factor β–induced increase in TCA cycle activity by diverting excess mitochondrial redox potential into the production of proline to support the translation of collagens (17).

Here we report the results of a small-scale screening campaign to identify proline analog inhibitors of human PYCR1. Twenty-seven commercially available compounds were screened using X-ray crystallography and enzyme kinetics assays. Five inhibitors of PYCR1 were found: l-tetrahydro-2-furoic acid (THFA), cyclopentanecarboxylate (CPC), l-thiazolidine-4-carboxylate (l-T4C), l-thiazolidine-2-carboxylate (l-T2C), and *N*-formyl l-proline (NFLP). The inhibition constants (*K_i_*) range from 100 μM for NFLP to 2 mm for THFA. The crystal structures of PYCR1 complexed with the inhibitors were determined at 1.80–2.35 Å resolution. The binding of NFLP is accompanied by protein conformational changes required to accommodate the inhibitor formyl group, including a 1-Å shift of an active site α-helix and the rotation of two side chains. The higher affinity of NFLP may be because of unique hydrogen bonds involving the formyl group. Moreover, NFLP induced proline accumulation and impaired proline biosynthesis as well as spheroidal growth in MCF10A H-RAS^V12^ breast cancer cells, which have been previously shown to rely on the proline cycle. In summary, our work generated the first validated chemical probe of PYCR1 and demonstrated proof-of-concept for screening proline analogs to discover inhibitors of PYCR1.

## Results

### Kinetic data for the inhibition of PYCR1

**Figure 2:**
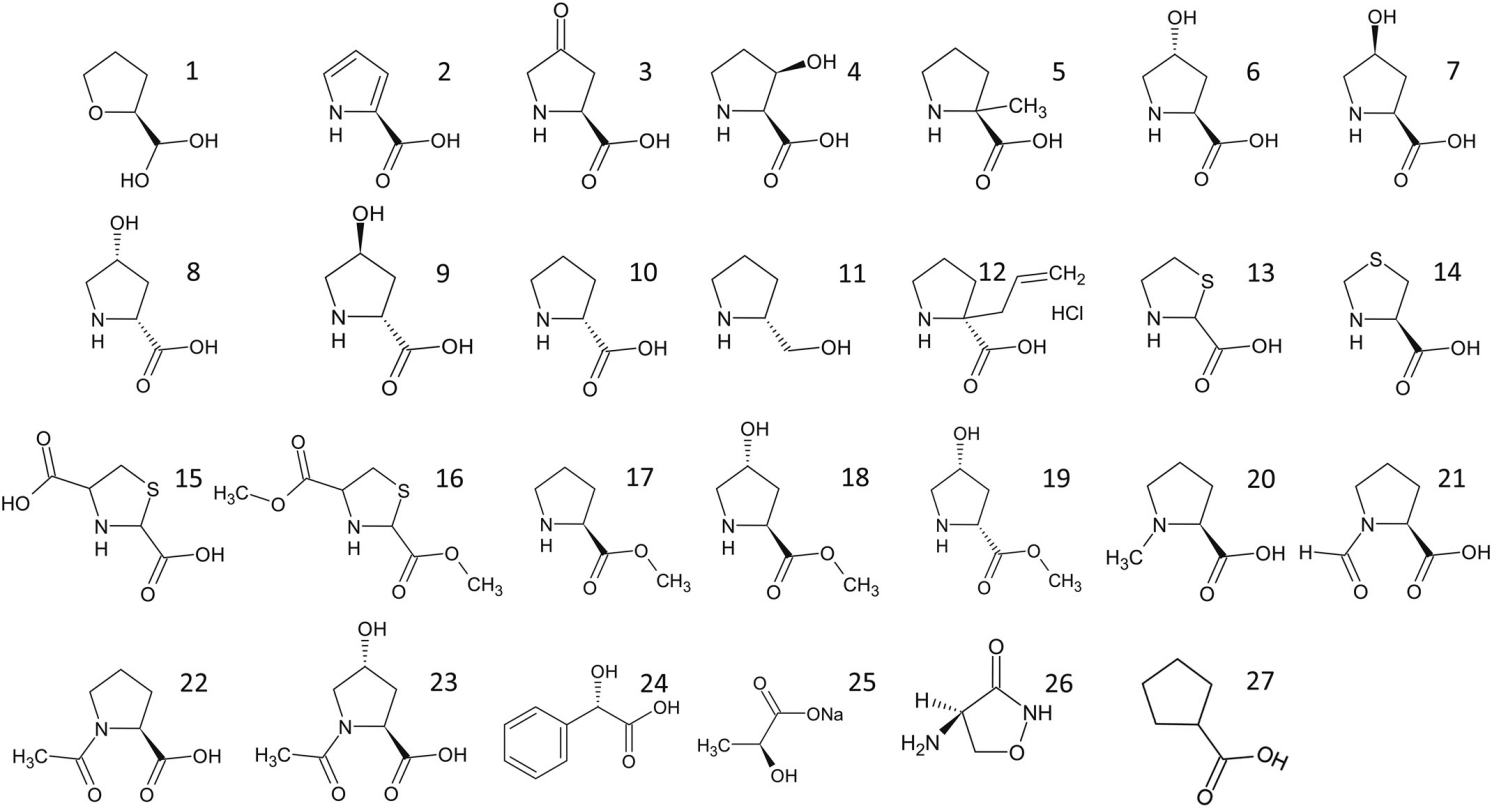
**Structures of the proline analogs screened *in crystallo* against PYCR1.***1*, (S)-(−)-tetrahydro-2-furoic acid; *2*, pyrrole-2-carboxylic acid; *3*, 4-oxo-l-proline, *4*, *cis*-l-3-hydroxyproline; *5*, α-methyl-l-proline; *6*, *trans*-4-hydroxy-l-proline; *7*, *cis*-4-hydroxy-l-proline; *8*, *cis*-4-hydroxy-d-proline; *9*, *trans*-4-hydroxy-d-proline; *10*, d-proline; *11*, R-(−)-2-pyrrolidinemethanol; *12*, (S)-α-allyl-proline; *13*, thiazolidine-2-carboxylate; *14*, l-thiazolidine-4-carboxylate; *15*, 1,3-thiazolidine-2,4-dicarboxylate; *16*, dimethyl 1,3-thiazolidine-2,4-dicarboxylate; *17*, l-proline methyl ester; *18*, l-4-hydroxyproline methyl ester; *19*, *cis*-4-hydroxy-d-proline methyl ester; *20*, *N*-methyl l-proline; *21*, *N*-formyl l-proline; *22*, *N*-acetyl l-proline; *23*, *trans*-1-acetyl-4-hydroxyl-l-proline; *24*, l-(+)-mandelic acid; *25*, sodium-l-lactate; *26*, d-cycloserine; *27*, cyclopentanecarboxylate.

**Figure 3:**
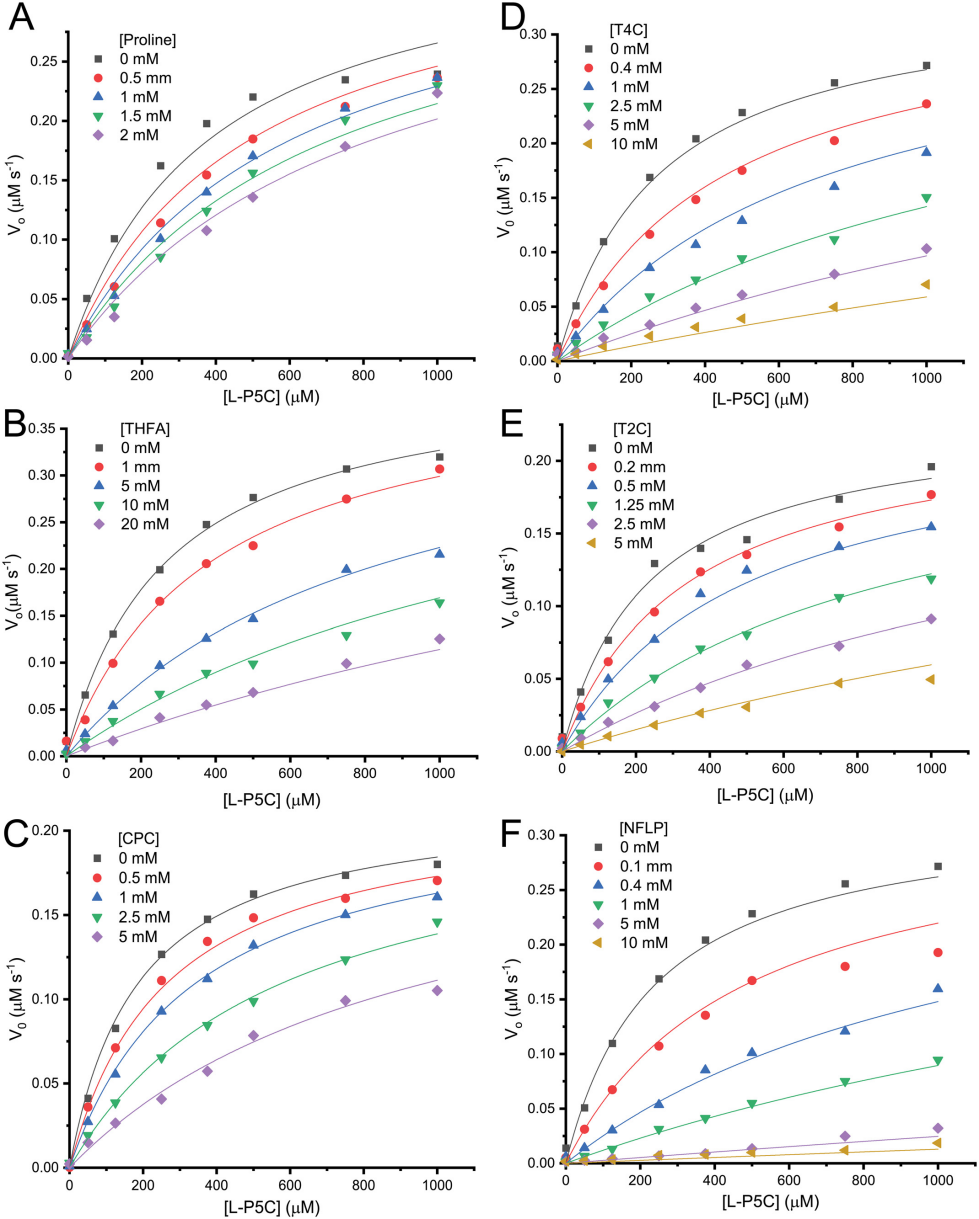
**Inhibition kinetics of PYCR1 with proline analogs.***A*, L-proline. *B*, THFA. *C*, CPC. *D*, T4C. *E*, T2C. *F*, NFLP. The data were measured in 50 mm Tris (pH 7.5) with 1 mm EDTA disodium salt while holding NADH fixed at 175 μm and varying d,l-P5C (0–2000 μm). The concentration of the substrate l-P5C was considered to be half the total d,l-P5C concentration added to the assays. Curves represent global fits of the data to a competitive inhibition model showing initial velocity in units of µm [NAD^+^] s^−1^ as a function of l-P5C concentration (µm).

Over two dozen proline analogs (Fig. 2) were screened using electron density from X-ray crystallography as the readout for binding to human PYCR1. These experiments suggested that THFA, CPC, l-T2C, l-T4C, and NFLP could be inhibitors of PYCR1. These initial hit compounds were validated using enzyme activity assays. Because the compounds bind in the P5C/proline site, the mechanism of inhibition was assumed to be competitive with P5C. Therefore, steady-state kinetic measurements were performed with L-P5C as the variable substrate (0-1000 μm) and NADH fixed (175 μm) (Fig. 3). For each compound, the data were fit globally to a competitive inhibition model (Equation 1) to obtain an inhibition constant, *K_i_*. The assays indicate that NFLP, l-T2C, and l-T4C are submillimolar competitive inhibitors of PYCR1 with estimated *K_i_* values of ∼100, 400, and 600 μm, respectively (Table 2). CPC and THFA are weaker inhibitors and have estimated *K_i_* values of 1 mm and 2 mm, respectively. For reference, we also determined the *K_i_* of the product l-proline to be 1.7 mm.

**Table 2:** Kinetic parameters for PYCR1 inhibition by proline analogs^a^

Analog	*K_m_* (μm)	*k*_cat_ (s^−1^)	*k*_cat_/*K_m_* (m^−1^ s^−1^)	*K_i_* (μm)
THFA	265 ± 17	69 ± 1	2.6 ± 0.5 × 10^5^	2249 ± 121
l-Proline	374 ± 41	58 ± 3	1.5 ± 0.7 × 10^5^	1718 ± 224
CPC	185 ± 9.0	35 ± 1	1.8 ± 1.1 × 10^5^	1193 ± 61
l-T4C	269 ± 21	54 ± 2	2.0 ± 0.9 × 10^5^	598 ± 39
l-T2C	232 ± 17	37 ± 1	1.5 ± 0.5 × 10^5^	438 ± 29
NFLP	236 ± 21	52 ± 2	2.2 ± 0.9 × 10^5^	99 ± 7.7


a *K_m_* and *k*_cat_ are the kinetic parameters for l-P5C determined at fixed NADH concentration of 175 μm. *K_m_*, *k*_cat_, and *K_i_* were obtained using global fitting to the competitive inhibition model in Equation 1.

### Structural basis of inhibition

**Figure 4:**
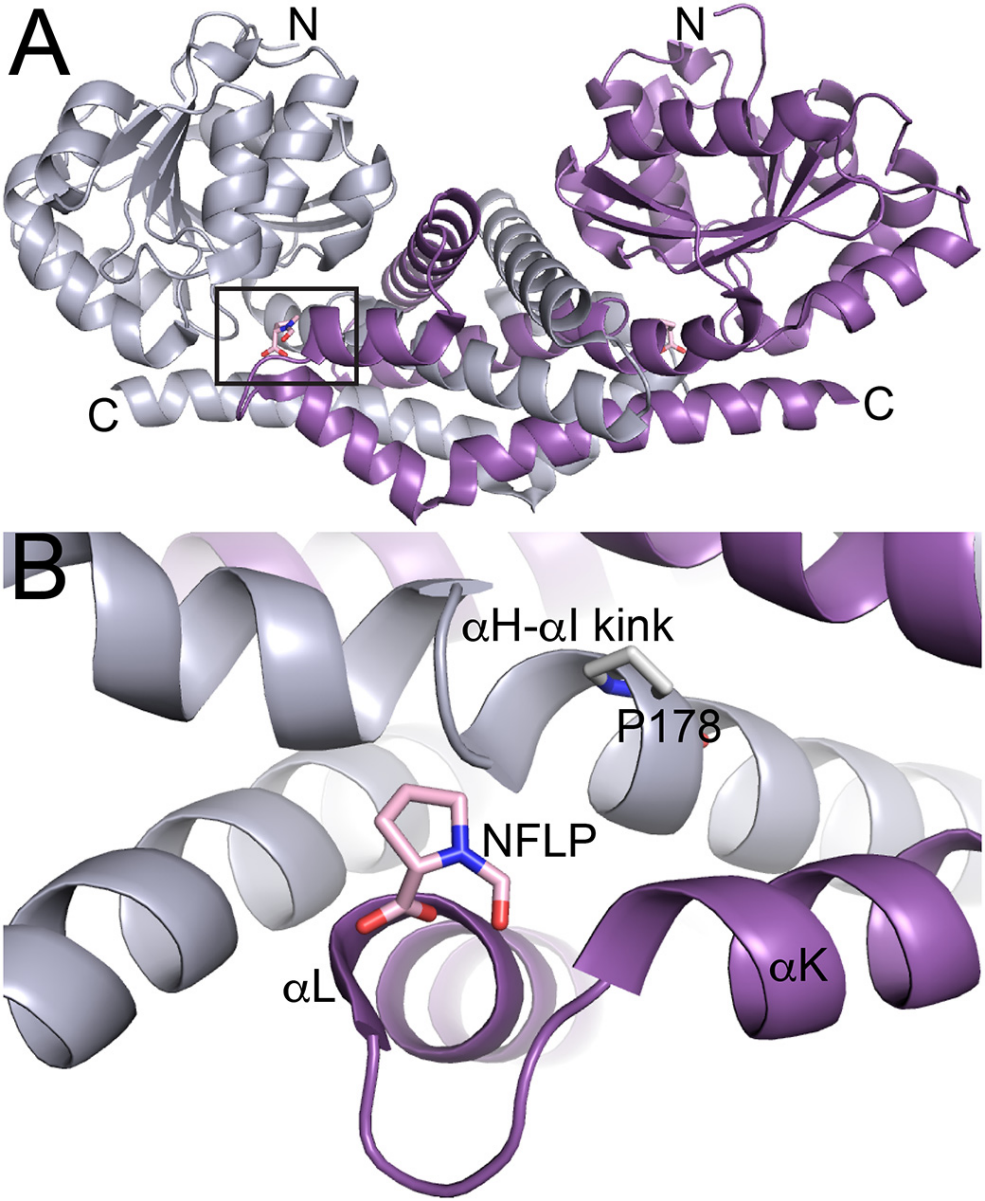
**Location of the inhibitor binding site.***A*, dimer of PYCR1 complexed with NFLP. The *box* indicates the inhibitor binding site. The two chains of the dimer have different colors. *B*, close-up view of the binding site. The two chains of the dimer have different colors.

**Figure 5:**
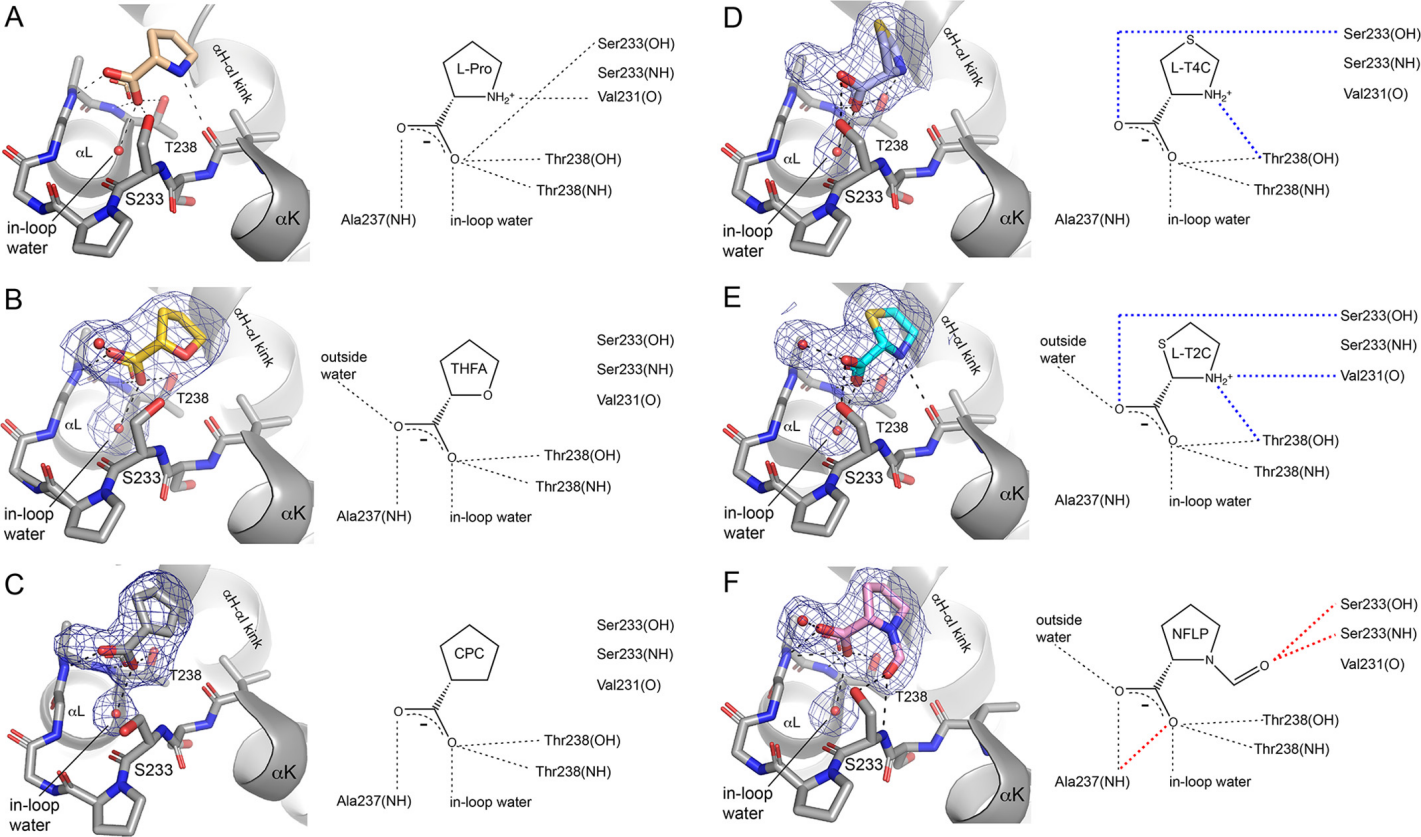
**The structures of PYCR1 complexed with proline analog inhibitors.***A*, l-proline (PDB code 5UAU). *B*, THFA. *C*, CPC. *D*, l-T4C. *E*, l-T2C. *F*, NFLP. The *blue cages* represent polder omit maps contoured at 4σ. In the schematic diagrams on the *right*, *blue dashes* denote hydrogen bonds unique to the thiazolidine complexes, and *red dashes* indicate those unique to the NFLP complex.

The crystal structures of human PYCR1 complexed with THFA, CPC, l-T4C, l-T2C, and NFLP were determined at high resolution limits of 1.75–2.35 Å (Table 1). Electron density for each analog was present in the previously characterized proline-binding site, which is located in a dimer interface and consists of the αK–αL loop of one protomer and a kinked α-helix of the other protomer (Fig. 4). The electron density maps for THFA and NFLP were unambiguous and allowed for modeling of the inhibitor at full occupancy in all five chains of the asymmetric unit (Fig. 5, *B* and *F*). In contrast, the interpretation of the electron density maps for l-T2C, l-T4C, and CPC was complicated by a sulfate ion binding to the active site, the occupancy of which varied from chain to chain. As a result, l-T2C was modeled only into chain A (refined occupancy of 0.87, Fig. 5*E*); l-T4C was modeled into chains A and E (occupancy of 1.0, Fig. 5*D*); and CPC was included in chains B and C (occupancy of 1.0, Fig. 5*C*).

**Table 1:** X-ray diffraction data collection and refinement statistics

	THFA	NFLP	T2C	T4C	CPC
Space group	C2	P2_1_2_1_2	P2_1_2_1_2	P2_1_2_1_2	C2
Unit cell parameters (Å,°)	*a* = 184.03*b* = 120.17*c* = 87.87β = 108.92	*a* = 164.63*b* = 88.51*c* = 115.46	*a* = 164.36*b* = 88.30*c* = 116.91	*a* = 163.16*b* = 88.00*c* = 115.79	*a* = 109.70*b* = 178.53*c* = 87.66β = 106.85
Beamline	ALS 4.2.2	APS 24-ID-E	APS 24-ID-E	ALS 4.2.2	ALS 4.2.2
Wavelength (Å)	1.0000	0.9792	0.9792	1.0000	0.9762
Resolution (Å)	60.1–2.35 (2.40–2.35)	82.3–1.95 (1.98–1.95)	95.3–1.75 (1.78–1.75)	49.2–2.30 (2.35–2.30)	47.2–1.93 (1.96–1.93)
Observations^a^	255,180	435,036	957,039	515,383	408,772
Unique reflections	72,417	120,287	170,953	74,689	116,598
*R*_merge_(*I*)^a^	0.082 (0.579)	0.080 (1.304)	0.051 (1.832)	0.091 (0.915)	0.055 (0.561)
*R*_meas_(*I*)^a^	0.096 (0.694)	0.094 (1.525)	0.056 (2.023)	0.099 (0.989)	0.066 (0.664)
*R*_pim_(*I*)^a^	0.051 (0.379)	0.048 (0.774)	0.023 (0.847)	0.038 (0.371)	0.035 (0.350)
Mean I/σ^a^	13.2 (2.0)	7.2 (0.8)	13.4 (0.9)	15.5 (2.2)	11.9 (1.8)
CC_1/2_^a^	0.997 (0.716)	0.996 (0.483)	0.999 (0.454)	0.998 (0.848)	0.997 (0.770)
Completeness (%)^a^	96.3 (75.2)	97.8 (99.7)	99.8 (99.9)	99.9 (100)	96.7 (97.6)
Multiplicity^a^	3.5 (3.1)	3.6 (3.8)	5.6 (5.6)	6.9 (7.1)	3.5 (3.5)
No. protein residues	1373	1374	1378	1377	1382
**No. atoms**					
Protein	9838	9926	10,025	9952	9887
Pro analog	40	50	8	16	16
Water	284	361	399	207	441
*R*_cryst_^a^	0.177 (0.244)	0.187 (0.312)	0.184 (0.349)	0.198 (0.223)	0.189 (0.318)
*R*_free_^a^	0.224 (0.340)	0.209 (0.375)	0.210 (0.381)	0.247 (0.286)	0.219 (0.348)
rmsd bonds (Å)	0.007	0.007	0.007	0.007	0.007
rmsd angles (°)	0.868	0.810	0.812	0.846	0.893
**Ramachandran plot**^b^					
Favored (%)	97.51	97.81	98.61	98.17	97.81
Outliers (%)	0.07	0.00	0.00	0.00	0.07
Clashscore (PR)^b^	2.94 (100)	2.05 (100)	2.38 (99)	3.35 (100)	3.18 (99)
MolProbity score (PR)^b^	1.51 (99)	1.29 (99)	1.24 (99)	1.46 (99)	1.52 (95)
**Average B (Å^2^)**					
Protein	46.7	52.3	53.4	49.7	35.0
Pro analog	42.5	51.5	45.5	48.2	36.0
Water	36.7	45.0	48.3	36.5	32.4
Coord. error (Å)^c^	0.30	0.25	0.23	0.27	0.22
**PDB code**	6XOZ	6XP0	6XP1	6XP2	6XP3


a Values for the outer resolution shell of data are given in parenthesis.

b From MolProbity. The percentile ranks (PR) for Clashscore and MolProbity score are given in parentheses.

c Maximum likelihood-based coordinate error estimate from PHENIX.

The binding poses of the inhibitors share some common features, which are also observed in the previously determined PYCR1-proline complex (18) (Fig. 5). In each case, the carboxylate of the analog binds in the αK–αL loop, while the ring contacts the kink between helices αH and αI of the opposite protomer of the dimer. The carboxylate of each inhibitor forms two hydrogen bonds with the side chain and backbone of Thr-238, plus a third hydrogen bond with a water molecule bound inside the αK–αL loop (“in-loop water”). Three of the analogs clearly formed hydrogen bonds to another water outside of the loop (THFA, l-T2C, and NFLP). It is possible that l-T4C also forms this hydrogen bond; however, the interpretation of the electron density for this potential interaction was complicated by the possible partial occupancy of sulfate.

The thiazolidine complexes are distinguished by hydrogen bonds not found in the other complexes (*blue dashes* in Fig. 5, *D* and *E*). The amino groups of l-T2C and l-T4C form a hydrogen bond with the hydroxyl of Thr-238, whereas the carboxylates hydrogen bond with Ser-233. These extra interactions are enabled by a subtle twisting of the thiazolidines compared with THFA and NFLP. Note the amino group of l-T2C also hydrogen bonds with the carbonyl of Val-231 (Fig. 5*E*), an interaction also observed in the proline complex (Fig. 5*A*).

NFLP also forms interactions not found in the other complexes (*red dashes* in Fig. 5*F*). The formyl group hydrogen bonds with the side chain and main chain of Ser-233, whereas the carboxylate forms a bidentate hydrogen bond interaction with Ala-237.

**Figure 6:**
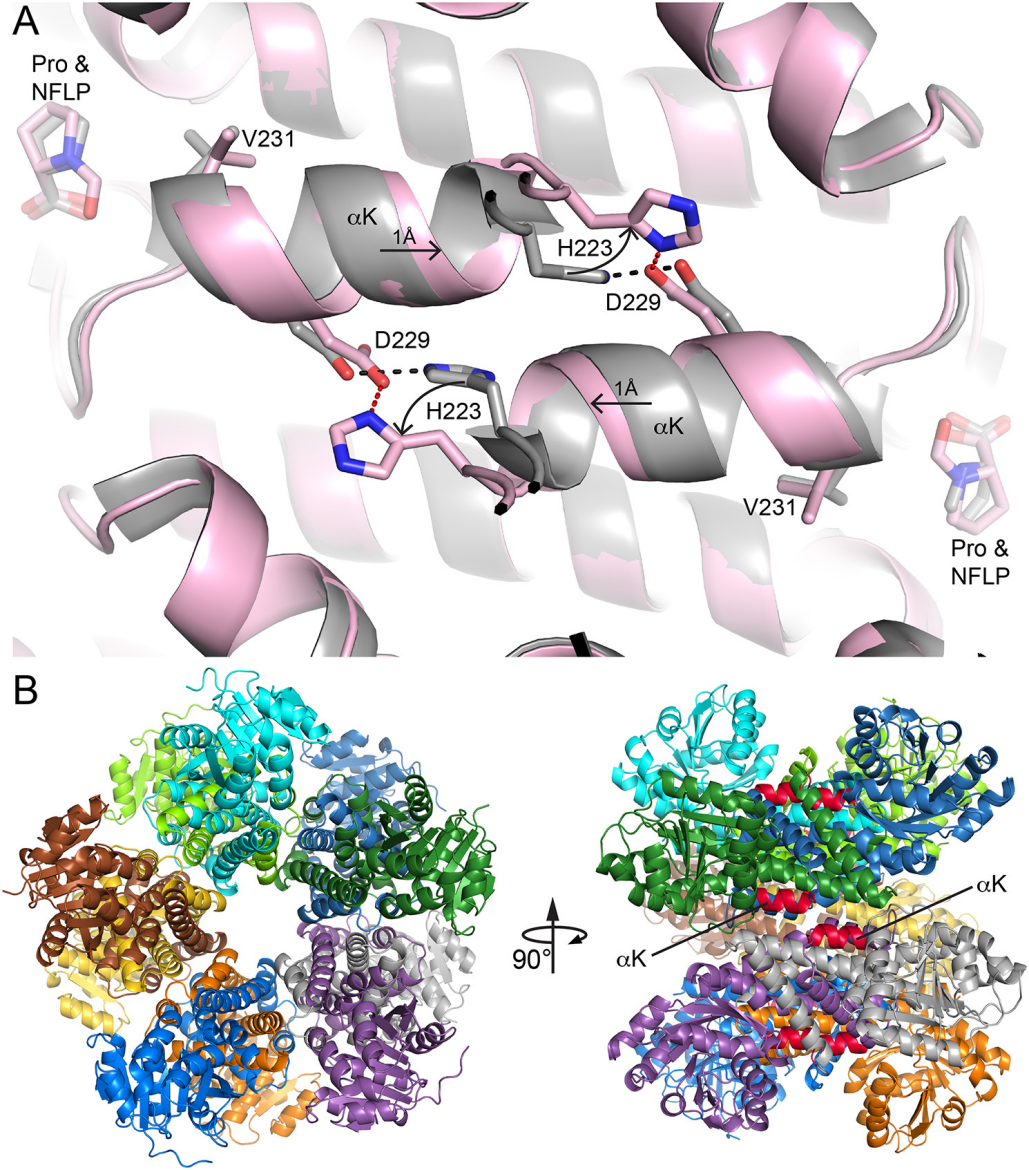
**Conformational changes needed to accommodate the formyl group of NFLP.***A*, superposition of the PYCR1-NFLP complex (*pink*) and the PYCR1-proline complex (*gray*) (PDB 5UAU). The *arrows* denote the directions of conformational changes needed to accommodate the steric bulk of the formyl group of NFLP. *Red and black dashes* denote the His-223–Asp-229 ion pairs in the NFLP and proline complexes, respectively. *B*, the location of αK in the decamer. *Left,* the decamer viewed down the 5-fold axis with each chain colored differently. *Right*, side view of the decamer with αK in *red*.

A unique feature of the NFLP complex is that the protein changes conformation to accommodate the formyl group of the inhibitor. This effect can be appreciated by comparing the structures of PYCR1 complexed with NFLP and proline (PDB ID 5UAU) (Fig. 6*A*). In the absence of conformational changes, the canonical proline binding pose places the formyl group 2 Å from the carbonyl O atom of Val-231 of αK. To avoid this steric clash, the side chain of Val-231 rotates from rotamer 1 to rotamer 2, and the αK helix translates by ∼1 Å (Fig. 6*A*). Because the αK helices of different protomers meet in an oligomer interface of the decamer (Fig. 6*B*), this conformational change involves two αK helices sliding past one another in opposite directions. This motion necessitates the rotation of the helix end-capping residue, His-223, to avoid steric clash with Asp-229 from another chain. Through these conformational changes, the formyl group of NFLP is accommodated in the active site while maintaining the His-223–Asp-229 intersubunit ion pair.

### Activity of NFLP in breast cancer cells

**Figure 7:**
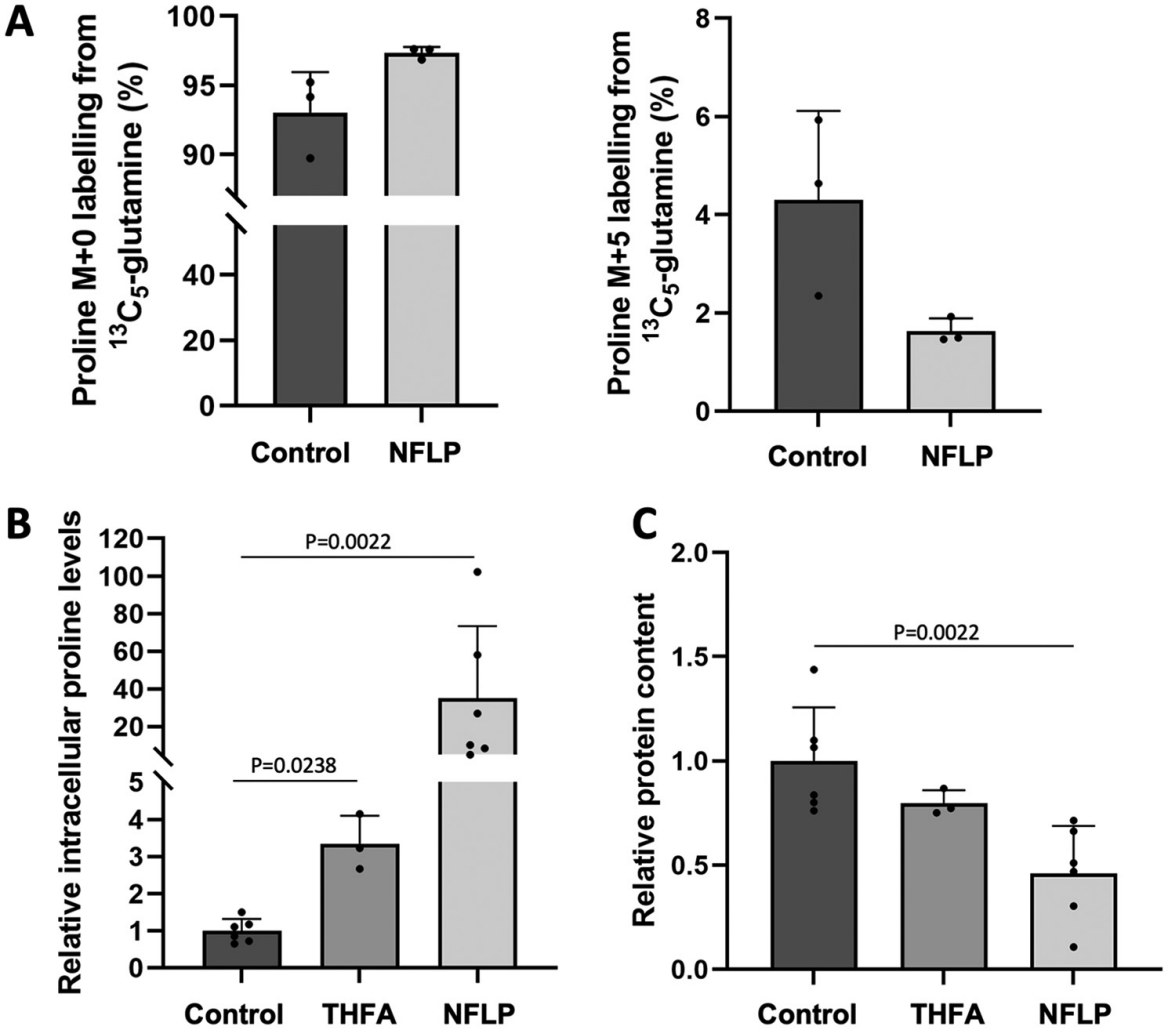
**NFLP targets proline metabolism in breast cancer spheroids.***A*, abundance of proline M + 0 and M + 5 labeling in MCF10A hRAS^V12^ spheroids upon ^13^C_5_-glutamine incubation and treatment without (*n* = 3) or with NFLP (5 mm; *n* = 3). Analysis was performed at 5th day of treatment. *B*, relative abundance of intracellular proline levels in MCF10A hRAS^V12^ spheroids upon treatment (5 days) without (*n* = 6), with THFA (*n* = 3) or with NFLP (*n* = 6). *C*, protein content in MCF10A hRAS^V12^ spheroids treated for 5 days without (*n* = 6), with THFA (*n* = 3) or with NFLP (*n* = 6). Bar graphs show mean ± S.D. from biological independent samples and *P*-values were obtained with Mann-Whitney tests.

We tested whether NFLP treatment decreases proline biosynthesis in breast cancer cells grown as spheroids. To do so we applied ^13^C tracer analysis (19), which is a method that can inform about nutrient contribution to biosynthetic pathways, to MCF10A hRAS^V12^ breast cancer spheroids grown in DMEM/F12 containing proline. Specifically, we measured the contribution of ^13^C_5_-glutamine to proline, which enables us to conclude on changes in *de novo* proline biosynthesis. In line with the results described above, we found that NFLP increased the unlabeled (M + 0) and decreased the ^13^C-labeled (M + 5) fraction of proline (Fig. 7*A*). This indicates that NFLP inhibits *de novo* proline biosynthesis.

Inhibition of proline metabolism has been previously shown to impair spheroid growth (5) and clonicity in cancer cells (11). These phenotypic changes were associated with elevated intracellular proline abundance, when either *PYCR1* was silenced or cancer cells were treated with the PRODH inhibitor THFA (5). Based on these previous findings, we decided to test whether NFLP also induced similar metabolomic and phenotypic changes. Thus, we measured proline abundance in MCF10A hRAS^V12^ spheroids treated with either NFLP or THFA. We observed that NFLP increased intracellular proline abundance by almost 40-fold compared with control and by about 10-fold compared with THFA (Fig. 7*B*). Next, we assessed spheroid growth based on protein content in MCF10A hRAS^V12^ treated with or without NFLP or THFA. In line with the metabolomic results we found that NFLP reduced spheroid growth based on protein content by 50% compared with control (Fig. 7*C*). Thus, we concluded that NFLP impairs MCF10A hRAS^V12^ breast cancer spheroids by targeting proline metabolism.

## Discussion

As evidence for the involvement of the proline cycle in cancer metabolism grows, so does the need for small-molecule inhibitors of the cycle as chemical probes. Currently, validated probes with well-defined mechanisms of action are available only for PRODH. These include the noncovalent inhibitor THFA and the covalent mechanism–based inactivator *N*-propargylglycine. It was previously shown that THFA impairs both spheroidal growth of breast cancer cells and metastasis formation in breast cancer mouse models (5), and it was recently reported that *N*-propargylglycine has anticancer activity in breast cancer cells, especially when used in combination with inhibitors targeting different proteins (21). These studies motivate chemical probe development targeting the proline cycle.

Here we used a “focused target-specific” (also known as “knowledge-based”) screening approach to identify inhibitors of PYCR1. Focused screening involves selecting from chemical libraries smaller subsets of molecules that are likely to have activity at the target protein based on knowledge of the target protein and chemical classes that have activity at the target (22, 23, 24, 25). This approach, applied at small scale, generated several hits and led to NFLP, a chemical probe exhibiting 100 μm inhibition of the purified enzyme and activity in breast cancer cells. These results establish proof-of-concept for screening proline analogs to discover chemical probes of PYCR1.

To our knowledge, NFLP is the first validated chemical probe of PYCR1. The mechanism of inhibition is competitive with P5C, as expected for a proline analog. The addition of the formyl group to proline increased the affinity by 17-fold, equating to ∼1.7 kcal/mol of binding free energy. The increased affinity is likely because of the extra hydrogens of the formyl group with the enzyme. This is supported by the observation that *N*-methyl-l-proline (*structure 20* in Fig. 2) was not an inhibitor. Interestingly, a 1-Å shift of the αK helix was needed to accommodate the formyl group. Apparently, local conformational changes in the substrate-binding loop did not occur to make space for the formyl group, indicating that the loop is rather rigid. Instead, longer range conformational changes occurred, which remodeled the decamer interface. Thus, it is possible that NFLP alters the self-association equilibrium of PYCR1. Additional studies focusing on the oligomeric state of PYCR1 in the presence of NFLP would further elucidate the disruption of the decamer interface and its role in inhibition. Interestingly, *N*-acetyl l-proline (*structure 22* in Fig. 2) also did not inhibit PYCR1, suggesting the 1-Å shift observed in the NFLP complex structure may be an upper limit, preventing the active site from accommodating larger substitutions at the amine.

NFLP phenocopies the *PYCR1* knockdown in breast cancer cells. As with the shRNA knockdown of the *PYCR1* gene (5), the treatment of MCF10A H-RAS^V12^ breast cancer cells with NFLP increased proline levels and decreased spheroid growth. The increase in proline in the knockdown suggests a decrease in PRODH activity, which indicated that P5C recycling by PYCR1 is important for sustaining PRODH activity (Fig. 1*A*). Presumably, the chemical inhibition of PYCR1 by NFLP also decreased proline cycle activity. Altogether, these results suggest that the observed in *cellulo* effects of NFLP are due to the on-target inhibition of PYCR1.

**Figure 8:**
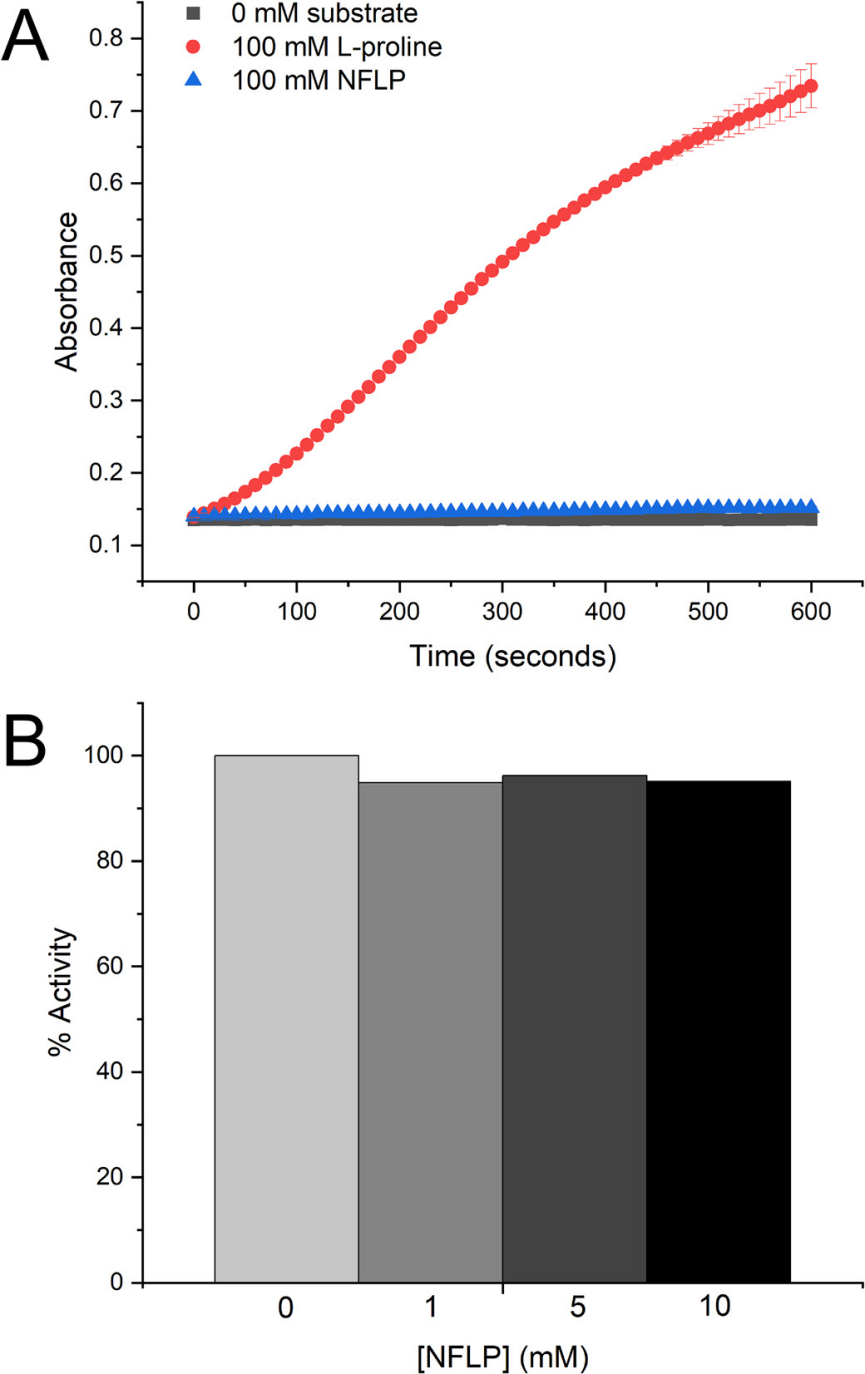
**Activity of a bacterial PRODH in the presence of NFLP.***A*, activity of the *E. coli* PutA PRODH domain with either 100 mm l-proline or 100 mm NFLP as the substrate. *B*, activity of the *E. coli* PutA PRODH domain with l-proline as the substrate (100 mm) at various NFLP concentrations. The rates have been normalized to the rate obtained in the absence of NFLP.

The other compounds analyzed here may have limited utility as chemical probes of PYCR1. For example, THFA and CPC are very weak inhibitors of PYCR1 (*K_i_* > 1 mm). We note that THFA is a known inhibitor of PRODH and has proven to be a useful chemical probe of that enzyme both *in cellulo* and *in vivo* (5, 21, 26, 27, 28). The observation that THFA only negligibly inhibits PYCR1 confirms that the effects of THFA in cells and *in vivo* are likely because of on-target inhibition of PRODH. The thiazolidines T2C and T4C are probably not useful for probing the proline cycle because they are oxidized by PRODH. We recently showed that T2C is rapidly oxidized by PRODH, and the oxidized species inactivates PRODH by covalently modifying the N5 of the FAD (29). T4C is a substrate for bacterial PRODH (30) and has been shown to interfere with l-proline metabolism and viability of the parasite *Trypanosoma cruzi* (31). Also, PYCR1 and some bacterial homologs have been reported to catalyze the NAD(P)^+^-dependent oxidation of T4C (reverse of the PYCR1 reductase reaction shown in Fig. 1*A*) (32, 33, 34). These other activities of T2C and T4C would need to be considered if the compounds were to be used as chemical probes of the proline cycle. In contrast, NFLP is neither a substrate nor an inhibitor of a bacterial homolog of human PRODH (Fig. 8), although we admit that these experiments cannot rule out the possibility that NFLP does impact human PRODH.

Milne *et al.* (35) recently reported the results of screening a library of 1280 pharmacologically active compounds against PYCR1. This effort identified pargyline as a potential inhibitor with a reported IC_50_ of 200 μm. About 60 derivatives of pargyline were synthesized and tested, resulting in a brominated pargyline compound with a reported IC_50_ of 10 μm, which also apparently inhibited proline biosynthesis from glutamine in human breast cancer cells (SUM-159-PT). However, dose-response curves for pargyline and its derivatives inhibiting purified PYCR1 were not shown, and the kinetic mechanism of inhibition was not determined. Given the dissimilarity of pargyline to P5C and NADPH, the mechanism of action of pargyline with PYCR1 is unclear and needs to be further investigated.

In summary, a small-scale focused screening strategy generated a validated probe of PYCR1. NFLP inhibits the purified enzyme with *K_i_* of 100 μm. X-ray crystallography revealed that NFLP occupies the P5C binding site, consistent with kinetic data showing the mechanism of inhibition is competitive with P5C. NFLP phenocopies the *PYCR1* knockdown in breast cancer cells by increasing proline levels and reducing spheroid growth. We suggest that NFLP will be a useful chemical probe for investigating the role of the proline cycle in cancer.

## Materials and methods

### Protein expression and purification

A C-terminal truncation variant of human PYCR1, which includes residues 1–300 of the full-length 319-residue enzyme, was used for kinetics and X-ray crystallography (18). PYCR1 was overexpressed in *Escherichia coli* BL21 (DE3) competent cells and purified using methods similar to those previously reported (18). Harvested cells were lysed via sonication in a mixture containing DNase I, lysozyme, and 0.2 mm phenylmethylsulfonyl fluoride protease inhibitor, and then the insoluble material was removed via centrifugation at 16,000 rpm for 1 h at 4°C. The resulting lysate was purified by gravity-flow chromatography on a column containing Ni^2+^–nitrilotriacetic acid resin (Qiagen) pre-equilibrated with binding buffer consisting of 50 mm HEPES, 300 mm NaCl, 10 mm imidazole, and 5% (w/v) glycerol at pH 7.8 (buffer A). The column was washed with buffer A supplemented with 30 mm imidazole, and then the bound protein was eluted with buffer A supplemented with 300 mm imidazole. Elution fractions containing PYCR1 were identified by SDS-PAGE and prepared for further purification by size-exclusion chromatography by dialyzing into 300 mm NaCl, 2% (w/v) glycerol, and 50 mm HEPES at pH 7.5. Size-exclusion chromatography was performed on a HiLoad 16/600 Superdex 200 column equilibrated with 50 mm HEPES, pH 7.8, 300 mm NaCl, and 5% (w/v) glycerol. Purified PYCR1 was concentrated to 8 mg/ml and stored at 4°C for subsequent crystallization trials. The concentration of PYCR1 was determined using the BCA method (Pierce) with BSA as the standard and confirmed spectrophotometrically at 280 nm using a molar extinction coefficient of 4720 M^−1^ cm^−1^ (Abs. 0.1% = 0.139) calculated from the amino acid sequence using ProtParam (36). We note the low extinction coefficient is because of the absence of Trp in PYCR1.

### Crystallization of apo PYCR1 for ligand soaking

Crystallization conditions for PYCR1 were identified using Hampton Research Index Screen and Crystal Screen I and II reagents in a sitting drop format (CrystalEX microplates) using an Oryx8 crystallization robot (Douglas Instruments). Screening trials contained 30 μl reservoir volumes with drops formed by mixing 0.3 μl of the protein stock solution and 0.3 μl of reservoir solution. Further optimization was conducted in Cryschem M sitting drop plates, with 500 μl reservoir volumes and 3 μl drops containing a 1:1 ratio of protein to reservoir solution. Microcrystal solutions made from initial hits were used for streak seeding during optimization trials. Optimized orthorhombic crystals in the space group *P*2_1_2_1_2 were grown from reservoir solutions containing 250 mm Li_2_SO_4_, 19% (w/v) PEG 3350, and 0.1 M HEPES at pH 7.5. We note this is the same *P*2_1_2_1_2 crystal form reported previously (18), except that Li_2_SO_4_ was used here instead of Na_2_SO_4_. In addition, crystals with space group C2 were grown in high ionic strength conditions consisting of 3 M NaCl and 0.1 M HEPES at pH 7.5–8.0. We note that this crystal form was also used previously (18).

### Preparation of crystals of PYCR1-inhibitor complexes

X-ray crystallography was used to screen 26 proline analogs for binding to PYCR1 (Fig. 2, *compounds 1–26*). The inhibitors tested included l-tetrahydro-2-furoic acid (Sigma-Aldrich no. 527890), pyrrole-2-carboxylic acid (Sigma-Aldrich no. P73609), 4-oxo-l-proline (hydrobromide) (Sigma-Aldrich no. 710962), *cis*-l-3-hydroxyproline (Sigma-Aldrich no. CDS009161), α-methyl-l-proline (Sigma-Aldrich no. 17249), *trans*-4-hydroxy-l-proline (Sigma-Aldrich no. H3656), *cis*-4-hydroxy-l-proline (Sigma-Aldrich no. H1637), *cis*-4-hydroxy-d-proline (Sigma-Aldrich no. H5877), *trans*-4-hydroxy-d-proline (Sigma-Aldrich no. 702501), d-proline (Sigma-Aldrich no. 858919), R-(−)-2-pyrrolidinemethanol (Sigma-Aldrich no. 281697), (S)-α-allyl-proline (hydrochloride) (Sigma-Aldrich no. 06594), d,l-thiazolidine-2-carboxylate (Sigma-Aldrich no. 467995), l-thiazolidine-4-carboxylate (Sigma-Aldrich no. T27502), 1,3-thiazolidine-2,4-dicarboxylate (Sigma-Aldrich no. CDS000186), dimethyl 1,3-thiazolidine-2,4-dicarboxylate (Sigma-Aldrich no. CDS000184), l-proline methyl ester (hydrochloride) (Sigma-Aldrich no. 287067), l-4-hydroxyproline methyl ester (hydrochloride) (Sigma-Aldrich no. 30681), *cis*-4-hydroxy-d-proline methyl ester (hydrochloride) (Sigma-Aldrich no. CDS014940), *N*-methyl l-proline (Sigma-Aldrich no. M8021), *N*-formyl l-proline (Sigma-Aldrich no. 728071), *N*-acetyl l-proline (Sigma-Aldrich no. A0783), *trans*-1-acetyl-4-hydroxyl-l-proline (Sigma-Aldrich no. 441562), l-(+)-mandelic acid (Sigma-Aldrich no. 63460), sodium-l-lactate (Sigma-Aldrich no. 71718), d-cycloserine (Sigma-Aldrich no. C6880). The proline analogs were added to apo crystals during cryoprotection by soaking the crystals in the reservoir solution supplemented with cryoprotectant (PEG 200 for the PEG 3350 form; glycerol for the NaCl form) and then in the same cryobuffer supplemented with 25–500 mm of the proline analog. Following equilibration, the crystals were harvested and rapidly plunged into liquid nitrogen.

Electron density evidence for binding was observed for THFA, NFLP, l-T2C, and l-T4C; therefore, the structures of these complexes were determined. The complex of PYCR1 with THFA was obtained by soaking a C2 crystal (NaCl form) for 15 min in a cryo-buffer consisting of the reservoir solution (3 M NaCl and 0.1 M HEPES at pH 7.5–8.0) supplemented with 30% glycerol and 50 mm THFA, followed by flash-cooling in liquid nitrogen. Crystals of the NFLP complex were prepared by soaking *P*2_1_2_1_2 crystals in the reservoir solution (250 mm Li_2_SO_4_, 19% (w/v) PEG 3350, 0.1 M HEPES, pH 7.5) supplemented with 20% (v/v) PEG 200 and 100 mm NFLP. The complexes with l-T2C and l-T4C were prepared similarly, except the Li_2_SO_4_ concentration was reduced to 25 mm and 50 mm of either l-T4C or d,l-T2C was included. Lowering the Li_2_SO_4_ concentration helped decrease the occupancy of sulfate ion in the active site, which made the electron density for the proline analog easier to interpret.

Crystals of PYCR1 complexed with CPC (Sigma-Aldrich no. C112003) were grown by co-crystallization (10 mm CPC) with a reservoir containing 200 mm Li_2_SO_4_, 18% (w/v) PEG 3350, and 0.1 M HEPES at pH 7.5. These crystals were harvested after soaking for 15 min in the reservoir solution supplemented with 20% (v/v) PEG 200 and 100 mm CPC. Although the *P*2_1_2_1_2 crystallization recipe was used, the CPC complex crystallized in space group C2. Curiously, this C2 form is different from the one described above, but the same as one reported previously by another group (PDB ID 2IZZ).

### Structure determination and refinement

X-ray diffraction data were collected at the Advanced Photon Source beamline 24-ID-E using an Eiger-16 M detector and at the Advanced Light Source beamline 4.2.2 of using a Taurus-1 detector. The data were processed with *XDS* (37) and AIMLESS (38). The asymmetric units of all three crystal forms contain five chains, corresponding to one-half of the PYCR1 pentamer-of-dimers decamer. Data processing statistics are summarized in Table 1.

The starting model for crystallographic refinement in PHENIX (39) was obtained from an apo structure of PYCR1 having the appropriate space group (PDB ID 5UAX for the THFA complex; PDB ID 5UAU for the orthorhombic structures; PDB ID 2IZZ for the CPC complex). The *B*-factor model consisted of one TLS group per protein chain and isotropic *B*-factors for all nonhydrogen atoms. Iterative model building and manual adjustments were performed using COOT (40). The restraint files for proline analogs were generated in PHENIX eLBOW (41) based on either the PDB ligand code or coordinates downloaded from PubChem (42). The structures were validated using MolProbity (43) and the wwPDB validation service (44). Refinement statistics are summarized in Table 1.

### Kinetic measurements of PYCR1 activity

Kinetic measurements were performed in a BioTek Epoch 2 microplate spectrophotometer using Corning 96-well UV-transparent microplates. Activity assays were performed by monitoring the consumption of NADH at 340 nm (ε_340_ = 6220 M^−1^ cm^−1^) using P5C as the substrate. d,l-P5C was synthesized as described previously (45), quantified using *o*-aminobenzaldehyde (*o*-AB), and stored in 1 M HCl at 4°C. Neutralization of d,l-P5C to pH 7.5 was performed immediately prior to assays using 1 M Tris (pH 7.5) and 6 M NaOH. The concentration of the substrate l-P5C was assumed to be half the total d,l-P5C concentration added to the assays. Inhibition assays (200 μl total volume) were performed at room temperature in ∼50 mm Tris (pH 7.5) with 1 mm EDTA disodium salt while holding NADH fixed at the approximate *K_m_* value (175 μm), and varying d,l-P5C (0-2000 μm).

The following microplate protocol was employed. Substrate mixtures containing P5C, NADH, EDTA, and Tris buffer were prepared in a deep well block for each P5C concentration. Mixtures containing enzyme and various concentrations of the inhibitor were prepared in microcentrifuge tubes and incubated on ice in buffer containing 50 mm HEPES pH 7.8, 300 mm NaCl, and 5% (w/v) glycerol. The final concentration of PYCR1 in the assay was 6.25 nm. Twenty μl of the enzyme-analog mixture was added to microplate wells using a single-channel pipette, and then the reaction was initiated by addition of 180 μl of the substrate mixtures using a multi-channel pipettor. The reaction traces were followed for 35 min. All absorbance values were corrected to a path length of 1 cm. Rates were calculated from linear regression of absorbance data from the first 3–4 min and converted to units of μM NAD^+^/second using the NADH extinction coefficient ε_340_ = 6220 M^−1^ cm^−1^. Data were fitted globally to a competitive inhibition model (Equation 1) with Origin software, where *v* is the initial velocity, *V_max_* is the maximal velocity, [*S*] is the concentration of the substrate, *K_m_* is the substrate concentration at half-maximal velocity, [*I*] is the inhibitor concentration, and *K_i_* is the competitive inhibition constant.

(Eq. 1) $$v=\frac{V_{max}[S]}{K_{m}\left(1+\frac{\left[I\right]}{K_{i}}\right)+[S]}$$

### PRODH activity measurements

Assays were performed to test whether NFLP is a substrate or inhibitor of PRODH. Because of challenges in obtaining active recombinant human PRODH, which is an inner mitochondrial membrane protein, these experiments used the PRODH domain of the bifunctional proline catabolic enzyme, Proline Utilization A from *E. coli* (EcPutA86-630). The active sites of human and bacterial PutA PRODH domains are very highly conserved. For example, all residues that contact proline analog inhibitors in crystal structures of bacterial PRODHs are identically present in human PRODH, as we described previously (6). Thus, bacterial PRODHs are considered to be good surrogates for identifying inhibitors of human PRODH. Indeed, the inhibitors discovered using bacterial PRODHs show on-target activity in cancer cells and animal models of cancer (5, 21).

PRODH activity was measured in a BioTek Epoch 2 microplate spectrophotometer by monitoring P5C as the adduct formed by the reaction with *o*-AB at 443 nm. The assays were conducted at 25°C in 20 mm MOPS, pH 7.5, and 10 mm MgCl_2_ with 4 mm *o*-AB, 0.15 mm menadione (electron acceptor to re-oxidize the FAD), and 1.3 μm of EcPutA86-630. The enzyme mixture contained EcPutA86-630, *o*-AB, menadione, and buffer. The substrate and/or inhibitor were spotted on the plate and the addition of the enzyme mixture initiated the reaction. When testing the substrate capabilities of NFLP, either 100 mm l-proline or 100 mm NFLP was present. When testing the inhibitory capabilities of NFLP, 100 mm l-proline was present along with 0, 1, 5, or 10 mm NFLP. The pH of the stock solution of NFLP was adjusted with NaOH to match the assay buffer.

### Spheroid cell culture

MCF10A cells that express hRAS^V12^ (MCF10A hRAS^V12^) were generated as described previously by Elia *et al.* (5). MCF10A hRAS^V12^ were cultured in DMEM/F12 enriched with 5% horse serum, 1% penicillin (50 units/ml), 1% streptomycin (50 μg/ml), 0.5 μg/ml hydrocortisone, 100 ng/ml cholera toxin, 10 μg/ml insulin, and 20 ng/ml recombinant human EGF. The *in vitro* model that allows these cells to form three-dimensional spheroids was performed as described formerly (5, 46). A soft-agar growth culture was prepared with a base layer consisting of agar and culture medium in 6-well plates. MCF10A hRAS^V12^ cells were plated on top of the base agar layer at 15,000 cells per well in normal medium, or medium supplemented with ^13^C_5_-labeled glutamine (Sigma-Aldrich, 98%) and incubated at 37°C in a 5% CO_2_ incubator for a period of 5 days. All analyses were performed at this 5th day.

THFA and NFLP were supplemented (pH neutralized with NaOH) at day zero to the media of MCF10A H-RAS^V12^ cells at the concentration of 5 mm. Protein levels were detected with the use of a Pierce BSA protein assay kit (Thermo Scientific). All growth experiments were conducted with *n* ≥ 3 biological replicates.

### MS analysis

The quenching and metabolite extraction of the three-dimensional spheroids were performed as previously designed by Elia *et al.* and Van Gorsel *et al.* (5, 46). In brief, the samples were quenched using a buffer consisting of 60% methanol and 10 mm ammonium acetate in a dry ice–ethanol bath (−40°C). Next, the metabolites were extracted via the methanol/chloroform procedure, again in a dry ice–ethanol bath of −40°C, in which the three-dimensional spheroids were disrupted mechanically with a tissue lyser. The upper methanol phase, the intermediate protein layer, and the lower chloroform phase were collected separately for analysis of the polar metabolites, proteins, and fatty acids, respectively. Upon derivatization, the isotopologues were separated with GC (7890A GC system, Agilent Technologies, Santa Clara, CA, USA) together with MS (5975C Inert MS system, Agilent Technologies, Santa Clara, CA, USA) in splitless mode to obtain metabolite abundances and labeling patterns (20). For the data analysis, the metabolite distributions were extracted from the raw ion chromatograms with the use of MSD Chemstation Data Analysis and further processed by a specifically developed MATLAB script (5). The total ion counts were normalized to the internal standards norvaline and glutarate to calculate the relative abundances of the metabolites.

For the three-dimensional spheroid experiments, all statistical data analysis was performed with the use of GraphPad Prism 8 on *n* ≥ 3 biological replicates (one 6-well plate represents one replicate). A Mann-Whitney test was performed to obtain the *p* values shown in the figures. Data are presented as mean ± S.D., as stated in the figure legends.

## Data availability

Coordinates and structure factor amplitudes for the structures reported in this paper have been deposited in the Protein Data Bank under accession codes 6XOZ, 6XP0, 6XP1, 6XP2, and 6XP3. All remaining data are contained within the article.
